# Mathematical models for predicting the toxicity of micropollutant mixtures in water

**DOI:** 10.2478/aiht-2025-76-3976

**Published:** 2025-09-30

**Authors:** Josipa Papac Zjačić, Hrvoje Kušić, Ana Lončarić Božić

**Affiliations:** University of Zagreb Faculty of Chemical Engineering and Technology, Zagreb, Croatia; University North, Koprivnica, Croatia

**Keywords:** additive model, aquatic organisms, independent action model, machine learning method, pesticides, PFAS, pharmaceuticals, QSAR, aditivni model, farmaceutici, metoda strojnog učenja, model neovisnog djelovanja, pesticidi, PFAS, QSAR, vodeni organizmi

## Abstract

Water pollution caused by micropollutants has been a global issue for decades, prompting the scientific community and industry professionals to develop new and effective wastewater treatment methods. Understanding the interactions of these compounds in real water samples is particularly challenging, as they contain complex mixtures that may alter the mechanism of action and toxic effects of these compounds on aquatic organisms. To address such challenges, computational methods and mathematical models have been developed to complement experimental research and predict the toxicity of micropollutant mixtures in water. This narrative review summarises current literature on such mathematical models, including the concentration addition (CA), independent action model (IA), and their combinations to predict the toxicity of mixtures involving pharmaceuticals, pesticides, and perfluorinated compounds. We also discuss computational methods like quantitative structure-activity relationship (QSAR) modelling and machine learning (ML). While the CA and IA models provide basic frameworks for predicting toxicity in chemical mixtures, their practical application is often limited by the assumption of additivity and by the complexity of real water mixtures. QSAR and ML approaches, though promising, face challenges such as limited data availability, overfitting, and difficult interpretation. Future research should focus on enhancing model robustness, incorporating mechanistic data, and developing hybrid approaches that integrate experimental and computational methods to improve the reliability of toxicity predictions for complex environmental mixtures.

## Abstract (hr)

Onečišćenje voda mikroonečišćivalima globalni je problem već desetljećima, zbog čega znanstvena zajednica i struka nastoje razviti nove učinkovite metode pročišćavanja otpadnih voda. Osobito je izazovno proučavanje međudjelovanja takvih spojeva u vodenom okolišu s obzirom na to da su stvarni uzorci vode zapravo smjese različitih tvari koje mogu promijeniti način njihova djelovanja, pojačavajući ili smanjujući toksične učinke na organizme. Zbog toga se, uz eksperimentalna istraživanja, koristimo i računalnim metodama, uključujući razvoj matematičkih modela za predviđanje toksičnosti smjesa mikroonečišćivala u vodi. Ovaj narativni pregled sažima dostupnu literaturu o takvim matematičkim modelima, uključujući model dodavanja koncentracije (CA), model neovisnog djelovanja (IA) i njihove kombinacije za predviđanje toksičnosti smjesa, koje uključuju farmaceutske proizvode, pesticide i perfluorirane spojeve. Također, raspravljamo o računalnim metodama poput modeliranja kvantitativnog odnosa strukture i aktivnosti (QSAR) i strojnog učenja (ML). Iako CA i IA modeli nude temeljne okvire za predviđanje toksičnosti kemijskih smjesa, njihova praktična primjena često je ograničena pretpostavkama aditivnosti i složenosti stvarnih uzoraka vode. QSAR i ML pristupi, koliko god obećavajući, suočavaju se s izazovima kao što su ograničena dostupnost podataka, prekomjerna prilagodba i nedostatak objašnjenja. Buduća istraživanja trebala bi se usredotočiti na poboljšanje robusnosti modela, uključivanje mehanističkih podataka i razvoj hibridnih pristupa koji integriraju eksperimentalne i računalne metode kako bi se poboljšala pouzdanost predviđanja toksičnosti za složene okolišne smjese.

The term micropollutant refers to a diverse group of substances with a significant negative impact on the environment and human health even at micro-scale concentrations found in the environment. These mainly include pharmaceuticals, pesticides, heavy and semimetals, personal hygiene products, mycotoxins, cyanotoxins, nanoparticles, nano- and microplastics, and perfluorinated compounds. Conventional wastewater treatment systems are generally ineffective in their complete removal and degradation (1). The importance of researching micropollutant mixtures in the environment lies in the interactions between micropollutants in mixtures, which can produce enhanced (additive or synergistic) or reduced (antagonistic) effect on organisms with respect to individual components (2). Understanding these effects is essential for accurate risk assessment and the development of efficient pollution control and environmental protection strategies.

The EU Water Framework Directive (WFD) (3) lists 45 pollutants requiring regular monitoring in the environment, bodies of water in particular, to ensure accurate, comprehensive, and current data across the member states. As experimental approaches to determining pollutant effects on organisms often rely on unrealistically high concentrations often required for environmental risk assessment in laboratory settings (4), this approach can lead to inaccurate safety thresholds as it ignores natural fluctuations in resource availability, like food supply for organisms, which can amplify the toxicity of contaminants at lower, environmentally relevant concentrations (5). Because of this, such data are best complemented with mathematical models based on actual environmental exposure levels to establish more accurate safety thresholds.

## BACKGROUND

### Micropollutants and their mixtures in the environment

Because of their toxicity, persistence, and tendency to bioaccumulate, micropollutants pose a serious environmental threat, as conventional wastewater treatment plants (WWTPs) are often inadequate in design and performance to remove them completely (6, 7). The removal efficiency starts at 20–50 % in primary treatment, 30–70 % in secondary, to reach over 90 % in tertiary treatment, but tertiary technologies are rare, except for disinfection, and require further upgrading (8).

Environmental concentrations of pharmaceuticals, for example, range from 0.0001–1 μg/L (9, 10). In surface waters, they are usually below 0.1 μg/L and in treated effluents below 0.05 μg/L (11). One of the most common pharmaceuticals in the environment is diclofenac, to be listed as priority pollutant (12), considering that its concentrations in a variety of environmental samples worldwide range from 0.1 to 8 μg/L (13).

However, mixtures of pharmaceuticals in the environment, such as those of antibiotics, analgesics, hormones, and psychiatric drugs can have far greater negative effects on aquatic organisms than individual drugs, such as disruption of the behaviour, growth, and reproduction of aquatic organisms or the development of bacterial resistance (14,15,16,17).

Another group of micropollutants raising concern are pesticides. Like pharmaceuticals, pesticide mixtures in water can pose a far greater risk to aquatic ecosystems and organisms than their components alone by disrupting physiological functions, impairing reproduction, altering behaviour, and affecting growth and development (18,19,20). According to Wan et al. (21) such adverse effects occur even at field-realistic and environmentally relevant levels. The European Commission Drinking Water Directive (22) set maximum permissible concentrations of mixtures of pesticides and their degradation products to 0.1 and 0.5 mg/L, respectively.

The third group of environmental micropollutants of concern are per- and polyfluoroalkyl substances (PFAS), widely used in cleaning agents, dyes, and fire retardants. As they resist degradation not only by biological processes but also by oxidation, which is the basis of conventional wastewater treatment systems, they are often referred to as “forever chemicals”, whose environmental concentrations range from 1.4 to 34.6 μg/L (23,24,25). PFAS are known to bioaccumulate in organisms and to pose potential health risks, including developmental issues, liver damage, immune system suppression, and certain cancers (26, 27). These substances have been shown to impact fish, birds, and other wildlife, leading to potential long-term ecological consequences (28, 29). As they persist and biomagnify in the food chain, organisms at higher trophic levels are exposed to higher concentrations of these harmful substances (30).

### Interactions between micropollutants in mixtures

Interactions between substances in a mixture can lead to a new harmful effect or change the effect of one of the compounds in the mixture. An additive interaction, as described by Folt et al. (31), occurs when the total effect of substances in a mixture is equal to the sum of their individual effects. This is considered the baseline or reference point. A synergistic interaction is observed when the combined effect is greater than the expected additive effect, indicating that the substances amplify each other’s toxicity. In contrast, an antagonistic interaction occurs when the total effect is lower than expected, suggesting that one compound counteracts or reduces the effect of another.

While this classification is widely used, interpretations and applications of these terms can differ between fields. Côté et al. (32) critically examine the conventional definitions and identify their limitations, especially in biological and environmental contexts. According to their analysis, such labels often oversimplify complex and nonlinear interactions. Instead of replacing the terms entirely, they recommend treating additive interactions as a neutral reference, synergistic effects as potentially harmful nonlinear interactions, and antagonistic effects as potentially beneficial nonlinear interactions depending on the context.

Chou (33) further clarifies that interactions in mixtures are not always mutual. For example, one compound may enhance the toxicity of another (potentiation) without receiving enhancement itself, or one compound may reduce another’s effect (suppression) without being affected in return. This nuance highlights the complexity of mixture interactions beyond traditional synergy and antagonism.

Environmental literature tends to over-report synergies between compounds, often without clear definition or rigorous testing (32). In contrast, meta-analyses using stricter methods typically find that additive effects are more common and synergies relatively rare. Antagonisms are often overlooked, because it is counterintuitive that two compounds should result in less damage together.

Current water regulations are focused on individual micropollutants, and there is no consistent approach to monitoring and assessing the risks posed by their mixtures. The United States Environmental Protection Agency (US EPA) has sought to address this issue with the 2000 Supplementary Guidance for Conducting Health Risk Assessment of Chemical Mixtures (34), whereas the European Food Safety Authority (EFSA) has defined a framework for assessing potential combined effects of pollutant mixtures in food (35), which complements current EU regulatory requirements for assessing the effects of individual components. In 2011, the EU Scientific Committee on Health and Environmental Risks (36) issued an expert group opinion on the applicability of mathematical models, such as additive and quantitative structure-activity relationship (QSAR) to predict combined toxicity of pollutants, considering that experimental data on combined toxicities of mixtures are very limited. In addition, toxicity tests are often expensive, time-consuming and sometimes use small animals, which should be avoided where possible. For these reasons, computational methods (*in silico* approach) are a better choice (37).

## MODELS PREDICTING THE TOXICITY OF MIXTURES

Regulatory authorities often assume additive toxicity for chemical mixtures in the absence of clear evidence to the contrary (32, 38). However, this is not the rule, as interactions may lead to synergy or antagonism, and it is important to test the same data set on different models, using different approaches. In this review, we have singled out four models from around 200 review and research articles published between 2013 and 2023. The most common is the concentration addition (CA) model, followed by the independent action (IA) model, QSAR, and machine learning (ML) model.

### Concentration addition model

The CA (or additive) model is used to predict combined toxicity of chemical mixtures based on component toxicity data. It assumes additive effects of each chemical at their respective concentrations (32) and similar mode of action, so that one chemical dilutes another and can be substituted with another chemical in a certain amount (33). The CA model can be explained with the Loewe additivity equation [1], such as the following for a binary mixture of compounds A and B (39, 40):

[1], $$\frac{C_{A}}{{\mathrm{EC}}_{\mathrm{yA}}}+\frac{C_{B}}{{\mathrm{EC}}_{\mathrm{yB}}}=1$$

where *C_A_* and *C_B_* are specific concentrations of each compound *A* and *B* resulting in the effect *y*. *EC_yA_* and *EC_yB_* denote the corresponding effect of compounds *A* and *B* alone that would generate the same response y as the mixture. A sum <0.8 or >1.2 indicates respective synergistic or antagonistic deviation from the CA model.

**Figure 1:**
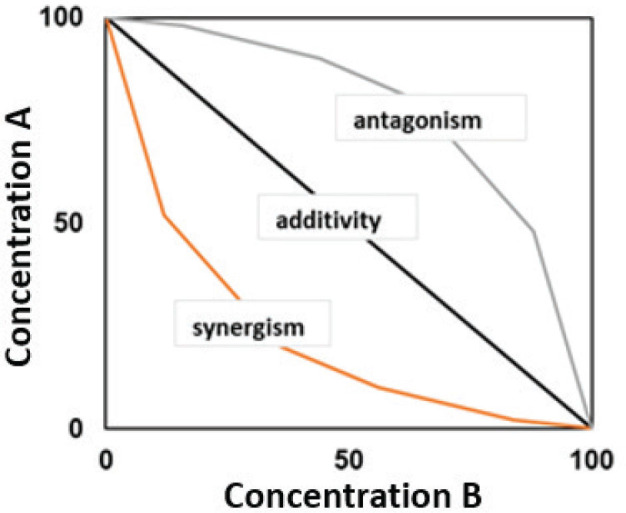
Isobologram of a binary mixture of components A and B

The toxicity behaviour of binary mixtures obtained with this model is most often illustrated by an isobologram (Figure 1), a two-dimensional plot with a straight line connecting the doses of each substance required to achieve the same effect individually. Substances are assumed to have the same or similar mechanism of action. If the combined doses are outside this line, the effect is either synergistic or antagonistic.

The CA model has often been applied in regulatory contexts and scientific studies. The 2013 EFSA guidance on tiered risk assessment, for instance, recommends the CA model as a key approach for evaluating pesticide mixtures (41), but its use has extended beyond them, encompassing pharmaceutical mixtures and industrial chemicals.

Several studies illustrate the advantages and reliability of the CA model in scenarios where chemicals act via the same mechanism. Belden et al. (42) applied it to assess the combined toxicity of chlorpyrifos and diazinon, organophosphate insecticides that inhibit acetylcholinesterase (AChE), in *Daphnia magna* and found that it accurately predicted additive toxicity based on the shared mode of action. Similarly, Puckowski et al. (43) used the CA model to evaluate the toxicity of flubendazole and fenbendazole, two anthelmintic pharmaceuticals, again observing additive effects in *D. magna*, consistent with CA predictions.

However, the CA model has notable limitations. It assumes a common mode of action of all mixture components, which does not always hold true. When chemicals affect different biological pathways, predictions based on simple additivity may be inaccurate. Cedergreen (44) has shown that combining chemicals with different mechanisms of action often leads to interactions that the CA model cannot predict. Similarly, Kortenkamp et al. (45) argue that the CA model overlooks non-additive interactions such as synergism or antagonism, which can lead to significant discrepancies in toxicity predictions. A case in point is the Schmuck et al. study (46), which, contrary to the expected additive effect, demonstrated an antagonistic effect of a tebuconazole (fungicide) and thiacloprid (insecticide) mixture in honeybees. Furthermore, although the CA model was originally intended for binary mixtures, it has increasingly been used for complex, multi-component mixtures, where its predictive power declines. Backhaus and Faust (47) caution that with more than two components, especially those with dissimilar actions or nonlinear interactions, the model often fails to provide accurate predictions.

Olwenn et al. (48) further support these concerns. Having analysed 1,220 experimental toxicity tests (two-thirds binary, one-third multi-component), they found that while the CA model fitted most mixtures, the occurrence of synergism or antagonism depended on both chemical concentration and mechanism of action. Taenzler et al. (49) investigated 47 pesticide mixtures in honeybee and found that additive toxicity was the most frequent outcome, yet some mixtures exhibited synergistic or antagonistic deviations, indicating that CA provides a good baseline but may overlook important interactions. These limitations highlight the need for more advanced modelling approaches when dealing with heterogeneous or environmentally relevant mixtures.

In conclusion, the CA model is a foundational and widely used approach for mixture toxicity prediction, particularly useful when components share similar mechanisms of action. It is a practical, conservative method for risk assessment but should be applied with caution when mechanisms differ or interactions are likely. In such cases, integrating CA with more complex or mechanistic models may yield more accurate and environmentally relevant toxicity predictions.

### Independent action model

In contrast to the CA model, the independent action (IA) assumes that components act independently, typically because they affect different biological targets or have different modes of action. Because of this, the IA model can complement the CA model with an alternative approach when no shared mechanism is assumed.

The combined effect is calculated as the sum of the effects of individual components in the mixture and their interactions, as follows:

[2], $$E=1-\prod i\left(1-e_{i}\right)$$

where *E* is the total effect of the mixture (e.g., mortality or inhibition rate), *e_i_* is the observed effect of the i-th compound when applied alone at the same concentration as in the mixture, and Π is the product across all compounds in the mixture. (50). The equation usually describes toxic effects of binary mixtures but can also explain multi-component mixtures.

The IA model is the most useful in assessing the toxicity of complex mixtures of pharmaceuticals, pesticides, PFAS, and other micropollutants in surface waters, sediments, and wastewater when these compounds are not expected to interact directly and when they exert their toxic effects via different physiological pathways.

While the CA model is often preferred in chemical risk assessment for its simplicity, it can fall short when extrapolating from high concentrations to environmentally relevant levels or when applied to mixtures with dissimilar mechanisms. In contrast, the IA model may offer improved accuracy under such conditions (51). Cedergreen et al. (52) tested both the CA and IA models on 98 mixtures of pesticides and pharmaceuticals across seven biological test systems. Their findings showed that the IA model alone accurately predicted approximately 20 % of the mixtures, CA about 10 %, and both models jointly predicted additional 20 %. However, around 50 % of mixtures were not adequately described by either model or their combination. Most deviations from model predictions were antagonistic. Strong synergistic effects, which are of greatest concern in risk assessments, were relatively rare, with the fungicide prochloraz identified as a notable exception. The authors therefore recommended that, in the absence of known synergists, CA can still offer conservative risk estimates within a reasonable margin of error even for mixtures with dissimilar components. Due to its lower data requirements, CA is suitable for individual-species assessments, whereas IA may be more appropriate for higher-level (e.g., multispecies or ecosystem) evaluations.

The IA model does, however, face several challenges. One of the main issues is the assumption that chemicals act independently without interacting and that their dose-response relationships are unaffected by the presence of other compounds. In practice, this assumption is often misplaced, as synergistic or antagonistic interactions may occur even when chemicals act on different biological targets. Moreover, the IA model requires high-quality, detailed dose-response data for each individual compound, which are often missing or incomplete, especially for environmental contaminants.

Shao et al. (53) provided experimental evidence of these limitations by showing strong antagonistic interactions between micropollutants in a ten-compound mixture in zebrafish (*Danio rerio*), highlighting that deviations from IA predictions can occur even in mixtures of seemingly unrelated substances. Similarly, Silva et al. (54) applied the IA model to assess the toxicity of a binary mixture of carbendazim and triclosan in *D. magna* across multiple biological endpoints. While some endpoints confirmed the IA-based additive predictions, others, particularly those related to genotoxicity, exhibited dose-dependent antagonism or synergism. These findings underline the importance of endpoint-specific evaluation in mixture toxicity assessments and demonstrate that the type of interaction may be related to the type of biological response.

In summary, the IA model is a valuable tool for modelling chemical mixtures with dissimilar modes of action, particularly in environmental contexts involving low-dose, multi-compound exposure. However, its assumptions of non-interaction and stable individual dose-response curves limit its applicability in many real-world cases. While IA can offer improved predictions in certain contexts, studies such as those by Cedergreen et al. (52), Shao et al. (53), and Silva et al. (54) show that it should not be used in isolation. Instead, IA should be applied alongside empirical data and other models, and chosen based on mixture characteristics and biological endpoints rather than theoretical fit alone.

### Alternative models based on CA and IA

While both CA and IA models assume additivity under their respective frameworks, they often yield comparable predictions, particularly when the individual dose-response curves exhibit similar slopes. Deviations from model predictions indicate potential synergism or antagonism, for which neither CA nor IA can fully account. These interactions are often illustrated with isobolograms; any significant departure from the central line denoting additivity signals that the model assumption is wrong. One way to establish a deviation from toxicity predicted by the two models is the mixture deviation ratio (MDR). MDR>1 indicates that the mixture is more toxic than predicted (synergism), while MDR<1 indicates weaker toxicity than predicted (antagonism). Belden (55) found that 34 % of pesticide mixture experiments on honeybees had MDR>2, indicating synergy, while MDR>5 was observed only in mixtures of azole fungicides and pyrethroids.

Furthermore, the choice of an appropriate null model can substantially influence how interactions such as synergy and antagonism are interpreted. Common frameworks like CA and IA are based on additive assumptions, which may not be suitable for all biological endpoints. For example, when mortality is the outcome, a multiplicative approach is often more appropriate. This is because once an organism dies from one compound, it cannot be affected again by another, meaning that the overall risk is better estimated by multiplying the probabilities of survival rather than adding the effects. Applying an additive model in such cases can distort the interpretation of interactions and lead to an overestimation of synergy.

To overcome this limitation, Chou (56) developed the combination index (CI) method for a quick and simple assessment of additivity, antagonism, or synergism in mixtures. This approach does not require prior knowledge of mechanisms of action of compounds. It is not a simple mathematical combination of CA and IA but does draw on the principles of both models using the following equation:

[3], $$\mathrm{CI}=\frac{D_{A}}{D_{A*}}+\frac{D_{B}}{D_{B*}}$$

where *D_A_* and *D_B_* are the doses of substances *A* and *B* in a mixture, and D_A*_ and D_B*_ are the doses of substances *A* and *B* required to achieve the same effect individually. This equation is derived from equation [1], since CI and CA are both based on Loewe additivity, and allows comparison between the combined and individual effects to determine if the substances interact in a synergistic (CI<1), additive (CI=1), or antagonistic (CI>1) manner. Unlike models that require assumptions about mechanisms of action, CI detects interactions based solely on the observed dose-response data and quantifies them on a scale from strong synergism to strong antagonism (Table 1). Just like the CA or IA isobolograms (Figure 1), those obtained with the CI model show synergy if the combined concentrations lie below the line connecting individual doses required to achieve the same effect, additivity if on the line, and antagonism above the line.

**Table 1:** Combination index ranges for five antibiotics in mixtures in terms of toxicity to photosynthetic aquatic organisms proposed by Chou (33)

**Combination index range**	**Description**
<0.1	Very strong synergism
0.10–0.30	Strong synergism
0.30–0.70	Synergism
0.70–0.85	Moderate synergism
0.85–0.90	Slight synergism
0.90–1.10	Nearly additive
1.10–1.20	Slight antagonism
1.20–1.45	Moderate antagonism
1.45–3.30	Antagonism
3.30–10	Strong antagonism
>10	Very strong antagonism

The method was tested by Ojo et al. (57) on PFAS mixtures in human liver cells (HepG2), who found predominantly low to medium synergistic effects (see Table 1). González-Pleiter et al. (58) used CI to assess the toxicity of individual and combined antibiotics in aquatic organisms and also found that synergism dominated, especially in mixtures with tetracycline. The CI model provided more accurate toxicity predictions than CA and IA, and risk assessment showed that the erythromycin-tetracycline combination posed a potential ecological risk.

Another alternative is the generalised concentration addition (GCA) model introduced by Howard and Webster (59). It is an extension of the traditional CA model that addresses its limitations. Namely, CA assumes that all mixture components have the same mode of action with parallel, full-efficacy dose-response curves, which is often not the case in real mixtures. Components can differ in maximal effect (e.g., full vs. partial agonists), have non-linear dose-response relationships, or act through different mechanisms yet produce a common biological outcome. Additionally, some chemicals may show no activity but still influence the overall mixture effect. The GCA model is calculated as follows:

[4], $$\sum_{i=1}^{n}\frac{c_{i}}{EC_{x,i}^{\mathrm{eff}}}=1$$

where *c_i_* is the concentration of component *i* in the mixture, $EC_{x,i}^{\mathrm{eff}}$ is the effective concentration of component *i* that would produce *x %* of the mixture’s maximal effect on its own, and *n* is the number of components in the mixture.

GCA accommodates diverse dose-response relationships and does not assume identical modes of action. It translates each component’s effect into an equivalent concentration of a reference compound, enabling additivity on the response scale and more realistic mixture modelling. This flexibility makes GCA especially useful in toxicology and pharmacology, notably for endocrine disruptors and receptor-mediated effects, where traditional CA falls short (60). Ultimately, GCA offers a more comprehensive and accurate framework for predicting mixture effects in complex real-world scenarios.

The superiority of the GCA model over traditional CA and IA has been demonstrated by Hadrup et al. (61). Using two chemical mixtures affecting hormone synthesis in H295R cells, they showed that GCA better predicted the effects on testosterone levels, especially in a mixture in which the chemicals had varying and limited efficacy. Although none of the models could predict the effects on oestradiol and progesterone due to opposing actions of the chemicals, the study clearly supports GCA as a more flexible and accurate tool for mixture toxicity prediction in complex, real-world scenarios.

Another complementary model to CA and IA for assessing the toxicity of chemical mixtures at low concentrations is the joint CA/IA mixture model described by Escher et al. (62). It is particularly useful in environmental settings, where many chemicals are present at low concentrations, at which dose-response relationships tend to be linear. Rather than serving as a true alternative to CA or IA, the joint model provides a supportive framework for estimating combined effects under conditions where CA and IA predictions often overlap, such as in complex mixtures of weak compounds. The formula to calculate the joint CA/IA model is as follows:

[5], $$E=\sum_{i=1}^{n}\left(\frac{c_{i}}{{\mathrm{EC}}_{i,\mathrm{low}}}\right)$$

where *E* is the combined effect of the mixture, *c****_i_*** is the concentration of the compound in the mixture, *EC_i, low_* the concentration of the compound that produces a low effect (typically at the lower end of the dose-response curve), and *n* is the number of compounds in the mixture. This approach is especially valuable for risk screening and prioritising mixtures for further testing.

Like CA and IA, the joint CA/IA model assumes additivity, but it simplifies prediction by summing contributions normalised to low-effect concentrations. It does not account for interactions between chemicals, making it best suited for complex mixtures where the effects are expected to be additive and interactions minimal.

Escher et al. (62) applied the joint CA/IA model to predict the cytotoxicity of 227 pesticide mixtures, each containing 2–17 compounds found in river water and agricultural leachates at low environmental concentrations. The model effectively predicted combined toxic effects when individual chemical effects were small and dose-response curves linear. The model is particularly suitable for environmental samples that typically require around a 10-fold enrichment before the effects become detectable, ensuring that predictions remain within the linear low-effect range. However, the joint CA/IA model assumes additivity and does not detect synergistic or antagonistic interactions.

### QSAR

QSAR modelling uses computational and statistical methods to predict the biological activity or toxicity of compounds based on their chemical structures without extensive experimental testing (63). Traditionally, it focuses on individual compounds, but recent advancements allow its application to complex mixtures.

Regardless of the variant, QSAR models process large sets of numerical descriptors – quantitative representations of chemical properties (e.g., octanol-water partition coefficient, acid dissociation constant, or molar refraction) – that have been obtained either experimentally or computationally through theoretical calculations. These descriptors capture key structural features and physicochemical properties relevant to toxicity. By analysing these data, QSAR models can identify patterns and relationships that help group chemicals with similar modes of action, facilitating mixture toxicity assessments and prioritising compounds for further study. QSAR methods range in complexity from simple linear models to advanced machine learning algorithms, depending on the problem and available data (64).

A specific variant of QSAR is the ecological structure-activity relationship (ECOSAR) model, which is tailored for predicting the environmental toxicity of chemicals on aquatic organisms such as fish, invertebrates, and algae. Made available to the public by the US EPA as a database-driven software tool (65), it complements the traditional QSAR models with rapid and efficient toxicity estimates using predefined chemical classes and built-in equations to estimate toxicity when experimental data are limited or unavailable. ECOSAR predicts key toxicity endpoints like the half maximal effective concentration (EC_50_) to screen large numbers of chemicals for potential environmental hazards. Its strength lies in correlating molecular structural features with toxicity, enabling the identification of chemical properties that contribute to environmental risks.

Graumans et al. (66) applied ECOSAR to assess the toxicity of pharmaceutical residues and their mixtures in treated wastewater and found that the effects were most often additive or synergistic. While advanced oxidation treatment reduced overall toxicity, transformation products of fluoxetine, cyclophosphamide, and acetaminophen showed increased toxicity. ECOSAR further predicted that prolonged thermal plasma oxidation would eventually render these transformation products less harmful.

The development of QSAR models begins with the selection of molecular descriptors and appropriate response variables. Both structural molecular descriptors and biological responses to them (action) can be calculated or based on conducted experiments from literature or databases. The collected data are then divided into two sets, that is, a validation set and a model building (training) set. Validation relies on statistical tools such as regression analysis to test the fit, that is the prediction accuracy of a developed model (67, 68). The higher the validation coefficient the better the fit of a model.

QSAR models are widely used to predict the toxicity of various pollutants, ranging from simple aromatic phenols to complex substances such as pharmaceuticals, pesticides, and PFAS (69, 70). Traditionally, these models assume that chemicals with similar molecular structures will exhibit similar toxicological effects, enabling compounds to be grouped by their likely mode of action (71). However, recent studies indicate that this assumption does not always hold, especially for complex mixtures where interactions between components can significantly alter toxicity outcomes (72,73,74).

Chatterjee et al. (72) exemplify the traditional QSAR approach, developing models based on 2D structural descriptors to predict the toxicity (EC_50_) of binary pesticide mixtures to *Photobacterium phosphoreum* and *Selenastrum capricornutum*. Their model effectively predicted mixture toxicity within the tested domain, relying mainly on structural similarity without explicitly incorporating interactions between mixture components.

Building on this, Quin et al. (73) developed a QSAR model for 45 binary and multi-component mixtures of pesticides and pharmaceuticals, targeting *Vibrio fischeri*. Their approach incorporated geometric and constitutional descriptors, including molecular volume, molecular weight, and specific carbon hybridisation (sp3/sp2), which proved key in predicting toxicity. Unlike simpler approaches, their model was able to capture both synergistic and antagonistic effects, showing better performance than traditional CA and IA models. This demonstrates how including additional descriptors and mixture-specific information can enhance QSAR predictions for complex mixtures beyond what basic structural similarity can achieve.

Wang et al. (74) proposed an integrated QSAR framework that combines mixture descriptors reflecting molecular interactions with established mixture toxicology models. Their extended Generalized Concentration Addition (XGCA) model was applied to binary mixtures of antibiotics and metal oxide nanoparticles tested on freshwater green algae. Unlike models grouping chemicals solely by structural similarity, the XGCA model accounts for combined effects based on shared modes of action and molecular interactions. It demonstrated improved accuracy over traditional models such as CA, IA, and standard GCA by better matching experimentally observed concentration-response curves across diverse mixture types. This approach underscores the value of integrating mechanistic and interaction-based descriptors within QSAR models to more realistically assess mixture toxicity.

A key challenge for QSAR in predicting mixture toxicity is incomplete or unknown information about the structure of mixtures and compound interactions that may not be fully captured by standard descriptors. To overcome this challenge, Toolaram et al. (75) developed a QSAR model that successfully identified several photolysis transformation products likely contributing to the toxicity of pharmaceuticals to V. fischeri. The study showed that focusing on key toxicophores such as the aromatic ring was sufficient to capture the toxic potential of these UV-degraded mixtures. This is highly relevant because it illustrates that even partly characterised transformation products can be included in mixture risk assessments using QSAR to achieve more realistic modelling of environmental samples containing unknown or poorly defined compounds.

Zhang et al. (76) developed a QSAR model for predicting PFAS concentrations that would not cause toxic effects (predetermined no-effect concentration, PNEC) in *Pseudokirchneriella subcapitata*, *Chlorella vulgaris*, *D. magna*, and *D. rerio*. The molecular descriptors from the US EPA database included internal molecular energy, translational kinetic energy, electron energy, vibrational interatomic energy, rotational energy, physicochemical parameters such as log_KOW_, and interactions between the highest occupied molecular orbital (HOMO) and lowest unoccupied molecular orbital (LUMO). Their model showed that logK_OW_ was the key parameter defining toxicity for this group of compounds.

In conclusion, QSAR modelling is a flexible and powerful approach for assessing mixture toxicity, though continued advancements are needed to address inherent challenges related to mixture complexity and incomplete chemical characterisation.

### Machine learning methods

Machine learning (ML) provides powerful tools for processing large datasets and identifying complex patterns between chemical features and biological responses. In toxicology, ML algorithms are increasingly used to develop predictive models that relate molecular descriptors to toxicity endpoints, enabling rapid, cost-effective screening of chemicals and their mixtures (77). This is particularly valuable in mixture toxicology where the number of possible chemical combinations far exceeds what can feasibly be tested experimentally.

ML methods are broadly categorised into supervised and unsupervised learning. Supervised methods rely on known input-output relationships derived from experimental data and are commonly used to classify toxicity outcomes or predict continuous toxicological responses (78). Unsupervised methods, on the other hand, detect hidden patterns in data without requiring predefined outcomes, helping to group chemicals by structural or activity similarity, which is useful for grouping similar mixtures based on their toxicological profiles.

Developing an ML model involves selecting a suitable algorithm and defining an appropriate molecular representation. The model is trained and validated with existing data, followed by external testing to evaluate its predictive robustness and applicability to new scenarios (79). In the context of mixture modelling, an additional challenge lies in defining suitable mixture descriptors that represent combined chemical properties effectively and account for potential interactions among components.

A notable example of large-scale ML application in toxicology is Tox21, a collaboration programme between several US agencies (80, 81). Although focused on single chemicals rather than mixtures, it demonstrates ML’s ability to handle vast datasets generated through high-throughput screening (HTS) of over 8,500 compounds, including pharmaceuticals, pesticides, and food additives. ML models trained on this dataset have produced millions of toxicity predictions, illustrating the scalability and insight of ML methods in regulatory toxicology and environmental risk assessment. The infrastructure and methodology developed through Tox21 provide a strong foundation for adapting ML to mixture toxicology as more complex datasets become available. However, despite the promise of these approaches, not all ML-based predictions have been validated experimentally, underscoring the ongoing need for external validation to ensure reliability.

Further advancing the application of ML to mixtures, Chatterjee et al. (82) developed and validated a QSAR model using ML techniques to predict the toxicity of 198 binary and multicomponent mixtures of pesticides and pharmaceuticals in *Aliivibrio fischeri*. The model, based on simple 2D molecular descriptors and partial least squares (PLS) regression, underwent rigorous cross-validation and demonstrated strong predictive accuracy. This highlights how ML-driven QSAR models can be applied specifically to mixtures and capture potential interactions between mixture components, which is an advantage over traditional mixture models that assume purely additive effects. The authors emphasised that careful mixture descriptor calculation and thorough validation are essential for ensuring model reliability in complex mixture scenarios.

A different but equally relevant application comes from Feinstein et al. (83), who used ML to evaluate the toxicity of 8,163 PFAS. They identified deep learning (DL), a subset of ML, as the most effective approach. Structural data from the US EPA’s PFAS database served as input for a transfer learning framework, allowing the DL model to leverage patterns learned from well-characterised compounds to predict toxicity in lesser-known ones. To address uncertainty in predictions, the authors employed techniques such as deep ensembles and a SelectiveNet model that abstains from low-confidence predictions. This approach illustrates how advanced algorithms can improve prediction reliability even in data-scarce domains.

In an environmental context, Cipullo et al. (84) applied two ML algorithms, neural networks (NN) and random forests (RF), to assess temporal changes in bioavailability and toxicity of complex mixtures in contaminated soils. These mixtures included petroleum hydrocarbons, heavy metals, and metalloids in soils enriched with compost or biochar. The NN model was used to provide continuous predictions of bioavailability over time, while the RF model helped identify which components and environmental conditions most influenced toxicity. This dual-model approach demonstrates how ML can effectively handle the temporal and compositional complexity of environmental mixtures, offering a more nuanced understanding of risk than traditional models. However, the authors also noted the need for broader validation across diverse soil types and contamination scenarios to improve generalisability.

Despite its potential, several challenges remain in applying ML to chemical mixtures. A major limitation is the scarcity of high-quality experimental toxicity data for mixtures, which hinders the training and validation of robust ML models. As shown by Feinstein et al. (83), even models that perform well may yield overconfident predictions in certain regions, making uncertainty quantification a critical consideration. Additionally, defining appropriate molecular descriptors for mixtures is more complex than for individual compounds. Chatterjee et al. (82) highlighted the importance of using appropriate mixture descriptor strategies such as additive rules to achieve predictive success, though such rules may still fail to capture all relevant interaction effects. Cipullo et al. (84) noted that models trained on a specific type of mixture or environmental matrix may not perform reliably when applied to different mixtures or contexts. Furthermore, while deep learning and neural networks offer high predictive power, their interpretability is limited, posing challenges for regulatory acceptance and mechanistic understanding.

Even with such challenges, ML holds great promise for enhancing chemical mixture assessment. By integrating structural information, environmental parameters, and biological responses, ML methods can model complex behaviour of mixtures in ways that traditional additive models cannot. Continued investment in high-quality mixture datasets, improved uncertainty estimation, and transparent model development will be essential to make the most of ML in mixture toxicology and environmental risk assessment.

## CONCLUSION

Accurate prediction of micropollutant mixture toxicity in aquatic environments necessitates a multifaceted approach. The CA model provides a conservative estimate for mixtures with similar mechanisms of action, while the IA model applies to those with distinct mechanisms. However, both models have inherent limitations that may lead to underestimation or overestimation of mixture toxicity. The CA model assumes that all components contribute additively to the overall toxicity, which may not hold true if components interact synergistically or antagonistically. Conversely, the IA model assumes independent action of components, which may underestimate toxicity when components interact.

Advanced models such as CI, GCA, and joint CA/IA offer more nuanced assessment by accounting for synergistic, antagonistic, or additive effects. The CI model, based on Loewe additivity, can identify and quantify interactions between components. The GCA model extends the CA approach by incorporating varying degrees of interaction. Joint CA/IA models integrate both approaches to assess mixtures of components with both similar and distinct mechanisms of action.

Further advancement was made possible with computational tools like QSAR and ECOSAR, which provide rapid, structure-based toxicity predictions, particularly valuable when empirical data are scarce. QSAR models have been successfully applied to predict the toxicity of pharmaceutical and pesticide mixtures, demonstrating their utility in environmental risk assessment. Similarly, ECOSAR models have been used to estimate the toxicity of various chemical mixtures, aiding in regulatory decision-making.

By analysing complex datasets and identifying patterns that traditional models might overlook, ML techniques further enhance predictive accuracy. ML models have successfully been applied to predict the toxicity of complex mixtures such as those of pharmaceuticals and PFAS, demonstrating their potential in environmental risk assessment.

Despite these advancements, challenges persist, including the scarcity of high-quality experimental data for mixtures, the complexity of defining appropriate mixture descriptors, and the need for robust uncertainty quantification. Addressing these issues through interdisciplinary collaboration and continued methodological development is essential for improving the reliability and applicability of toxicity predictions in complex environmental scenarios.
